# Evaluation of a digital FFQ using 24 h recalls as reference method, for assessment of habitual diet in women with South Asian origin in Norway

**DOI:** 10.1017/S1368980024000302

**Published:** 2024-02-06

**Authors:** Monica H Carlsen, Torunn Holm Totland, Radhika Kumar, Therese ML Lensnes, Archana Sharma, A Anita Suntharalingam, Anh Thi Tran, Kåre I Birkeland, Christine Sommer

**Affiliations:** 1 Department of Nutrition, Institute of Basic Medical Sciences, Faculty of Medicine, University of Oslo, Postboks 1046 Blindern, Oslo 0317, Norway; 2 Department of Physical Health and Ageing, Division of Mental and Physical Health, Norwegian Institute of Public Health, Oslo, Norway; 3 Department of Endocrinology, Akershus University Hospital, Lørenskog, Norway; 4 Department of Endocrinology, Morbid Obesity and Preventive Medicine, Oslo University Hospital, Oslo, Norway; 5 Institute of Clinical Medicine, Faculty of Medicine, University of Oslo, Oslo, Norway

**Keywords:** FFQ, Diet, South Asian immigrants, Method evaluation

## Abstract

**Objective:**

Dietary assessment tools should be designed for the target population. We developed an FFQ designed to assess diet in South Asian women in Norway. The study objective was to evaluate this FFQ using 24-h dietary recalls as reference method.

**Design:**

Approximately 3 weeks after the participants (*n* 40) had filled in the FFQ, the first of three non-consecutive 24-h dietary recalls was completed. The recalls were telephone-based, unannounced and performed by a trained dietitian, with 2–3 weeks between each interview.

**Setting:**

The DIASA 1 study, in Oslo, Norway.

**Participants:**

Women of South Asian ethnic origin participating in the DIASA 1 study were invited to participate in the evaluation study.

**Results:**

The WebFFQasia significantly overestimated the absolute intake of energy, protein, fat and carbohydrates compared with the 24-h dietary recalls. Absolute intakes of sugar, starch and fibre did not differ significantly between the methods. For energy percentages (E%), there were no significant differences, except for monounsaturated fat. Correlations were strong for E% from sugar and saturated fat and moderate for E% from fibre, carbohydrate, total fat and protein. Fourteen food groups out of twenty three were not significantly different compared with the reference method, and sixteen groups showed strong to moderate correlations.

**Conclusion:**

The WebFFQasia may be used to assess E% from habitual diet and can adequately estimate intakes and rank participants according to nutrient intake and main food categories at group level.

People of South Asian origin have an increased risk of gestational diabetes, type 2 diabetes and atherosclerotic CVD, often developed at a younger age than compared with other ethnic groups^(1–3)^. This increased susceptibility applies regardless of residence; both in their native countries and after immigration to western countries^(4)^. Overweight, obesity, unhealthy diet and physical inactivity are key risk factors for type 2 diabetes. A recent meta-analysis reported a relative risk reduction of 35 % for type 2 diabetes after diet and physical activity interventions^(5)^. Understanding the dietary habits of South Asian immigrants to western countries and their descendants is crucial for providing appropriate and targeted health care. However, assessing the diet of this population presents some challenges^(6)^. Recruitment of South Asians to participate in health research has been shown to be difficult^(6)^. Also, variations in nutrition-specific literacy may cause many traditional nutritional assessment methods difficult to use in parts of the population. In addition, FFQ developed for the general population may not accurately capture the diets of South Asian immigrants. Similarly, FFQ developed in their countries of origin do not reflect the changes in their diet after immigration^(6)^. Thus, there is a need for dietary assessment tools specifically designed for this population.

To address this issue, we recently evaluated the performance of a web-based FFQ designed for assessment of a traditional Norwegian diet, in South Asian women living in Norway, using three 24-h dietary recalls as a reference method^(7)^. The results showed this FFQ to adequately estimate the intakes of several food items and nutrients at the group level; however, estimates of protein, carbohydrates and dietary fat could be improved on. Based on the recipes gathered through the 24-h dietary recalls^(7)^, we developed a new version of the web-based FFQ specifically designed for individuals of South Asian origin living in Norway, the WebFFQasia.

The aim of the present study was to evaluate the performance of the WebFFQasia in a population of South Asian women living in Norway, using dietary information collected from three 24-h dietary recalls as reference method.

## Methods

### Study design and participants

The DIAbetes in South Asians (DIASA) 1 study included 179 women of South Asian (Pakistan, India, Sri Lanka and Bangladesh) and 108 women of Nordic (Norway, Sweden, Denmark, Finland and Iceland) origin, followed during specialist health care for gestational diabetes, 1–3 years before recruitment. The DIASA 1 study was performed between 1 September 2018 and 31 December 2021 at Oslo University Hospital, Akershus University Hospital and Drammen Hospital. A detailed description of the cohort has been published previously^(8)^. In brief, the DIASA 1 study investigates the impact of South Asian and Nordic ethnicity on the prevalence and characteristics of prediabetes and diabetes in women, 1–3 years after a pregnancy with gestational diabetes^(8–10)^. Eligible women received an invitation letter and a telephone invitation in their native language, and all participants signed study consent forms^(8)^. All women participating in DIASA 1 were invited to complete the WeFFQasia. From June 2021 to the middle of February 2022, women of South Asian ethnic origin who had completed the WebFFQasia were invited to participate in the evaluation study. Due to slow recruitment caused by the COVID-19 pandemic, we implemented retrospective recruitment, inviting participants who had already completed the WebFFQasia (>6 months in advance) from October 2021. These participants were asked to fill in the WebFFQasia questionnaire once more to participate. Exclusion criteria were non-Asian origin and a new pregnancy. We aimed at recruiting fifty participants^(11)^, based on the time frame and resources available in this project.

### Background data

Age was calculated from the date of birth. South Asian origin was defined as both parents born in Pakistan, India, Bangladesh or Sri Lanka. Height and weight were measured at the study sites, according to international standards^(12)^ and used to calculate the BMI. Height was measured to the nearest 0·1 cm by a fixed stadiometer, weight was measured to the nearest 0·1 kg by an electronic scale and BMI was calculated from the formula weight (kg)/height (m)^2^. The waist circumference was measured to the nearest 0·1 cm by a non-elastic measuring tape midway between the lower rib and the iliac crest, the participant standing and breathing normally, at exhalation. The hip circumference was measured at the trochanter region. Information about smoking and alcohol habits was collected through self-administered questionnaires in DIASA 1. Questions about education, living situation and work situation were asked during the evaluation study by the interviewer in the 24-h dietary recalls.

### Dietary methods

#### FFQ

The WebFFQasia is a web-based, self-administered, quantitative FFQ consisting of approximately 310 questions about habitual diet over the past year, including both South Asian and Norwegian dishes. The present WebFFQasia is a revised version of a previously validated original web-FFQ, designed to capture the habitual Norwegian diet, which includes 270 questions^(13)^. The changes and revisions made to the original web-FFQ, including the list of South Asian food items, were based on the results from a Master’s thesis conducted in 2018^(7)^. The Master’s project evaluated the performance of the original web-FFQ in women of South Asian origin and concluded that revisions should be made to improve estimates of nutrient, energy and food intake. Specifically, questions about curries, chapati, idiyappam, dosa and South Asian desserts should be included. The original web-FFQ was thus revised according to these recommendations. When filling in the WebFFQasia at home, the participants indicated whether their habitual diet was generally Norwegian or South Asian. All who indicated the latter were then directed to additional twenty-three questions about specific South Asian food items before they continued with the rest of the WebFFQasia, consisting of the original web-FFQ. If the respondent indicated a mostly Norwegian diet, she was automatically led to the questions that constitute the original FFQ in WebFFQasia. The WebFFQasia also included questions about dietary supplements. The questions are largely organised in meals and associated food groups, including twelve questions about bread, thirty four about bread spreads, forty one about beverages (including hot and cold, with and without alcohol and drinking and mineral water), forty nine about dinners (vegetarian, meat and fish dinners), forty two about fruit, vegetables, berries, nuts and seeds, twenty four about desserts, sweets and salty snacks, in addition to questions about condiments, sources of fat and dietary supplements. Frequency alternatives range from never, times per month, times per week, to several times per day. Portion sizes are given in household measures, or in pictures of portions. The WebFFQasia has a dynamic design and may be used on all digital platforms, including mobile phones. The participants were therefore asked what digital platform they used when filling in the FFQ. The food and nutrient composition database and calculation system ‘KostBeregningsSystem’^(14)^, database AE-22 (Department of Nutrition, University of Oslo) was used to estimate energy and nutrient intakes from the FFQ and 24-h dietary recalls.

#### The 24-h dietary recalls

Three 24-h dietary recalls were conducted on three non-consecutive days, including two weekdays and one weekend day. The first interview was conducted at least, and approximately, 3 weeks after the participants had completed the WebFFQasia. The interviews were unannounced and telephone based and carried out by a trained dietitian, with 2–3 weeks between each interview. The interview followed a standard protocol for 24-h dietary recalls, developed at the Department of Nutrition, University of Oslo. In brief, according to protocol, the interviewer goes through all the meals the participants had the day before, twice and asks additional questions with regards to foods and beverages that we know from experience are easy to forget. On average, an interview would take approximately 30 min. All food items were coded directly into the KostBeregningsSystem database during the interview. To assist in the estimation of amounts, the participants used a booklet with pictures of portion sizes and sizes of plates, bowls, glasses and cups. Of all the interviews conducted, two individual interviews, with two different participants, were conducted without the booklet, because it was not available at the time, and the participants therefore provided their dietary information in household measures (half a plate, one cup, etc.) instead. The trained dietitian who performed the interviews was multilingual and conducted the interviews in Norwegian or the participant’s preferred language (Urdu/Hindi/English). The dietitian was also familiar with the diet culture of South Asian populations in Norway.

## Statistics

All statistical analyses were performed using the IBM SPSS statistical software package, versions 26 and 29.0.0.0. The statistical significance level was set to *P* < 0·05 for all analyses. Continuous variables were assessed for distribution and are presented accordingly. Categorical data are presented as numbers (%). We used paired-sample Student’s *t* test or Wilcoxon’s signed-rank test to assess differences between the dietary methods, according to distribution. Pearson’s *r* and Spearman’s ρ were used to explore correlation. Correlations were defined as poor when <0·30, moderate between 0·30 and 0·49 and strong when >0·50^(15)^. To further evaluate the FFQ, cross-classification was used to compare quartiles of intake, Bland–Altman (BA) plots were used to assess systematic bias,^(16)^ and drop-line graphs were used to further visualise individual differences between methods.

## Results

### Study sample

Forty-two participants were included; however, one participant completed only one of the three 24-h dietary recalls and one recorded extreme and unrealistic levels of intake of several foods in the FFQ. Thus, data from forty participants were included in the analyses. Most participants were of Pakistani origin, followed by Indian and Sri Lankan origin (Table 1). Mean age was 35 years, range 25–42.

**Table 1:** Anthropometric and socio-demographic background of participants (*n* 40)

Variable	Mean	sd
Age (years)	35	4
Height (cm)	159	7
Weight (kg)	73	19
BMI (kg/m^2^)	29	6
	*n*	%
Education		
Elementary school	4	10
Secondary school	9	22·5
University level	27	67·5
Ever smoker	2	5·0
Intake of alcohol last year	5	12·5
Ethnic origin		
Pakistan	25	62·5
India	9	22·5
Sri Lanka	6	15·0
Lives with partner and children	39	97·5
Working situation		
Homemaker	8	20·0
Employed (position ≥50 %)	24	60·0
Unemployed	5	12·5
Other	3	7·5

Height and weight were measured at the study clinic. Position ≥50 %, employment part time, working hours 50 % or more.

### Relative evaluation of the FFQ

#### Intake of energy and energy-providing nutrients

**Fig. 1:**
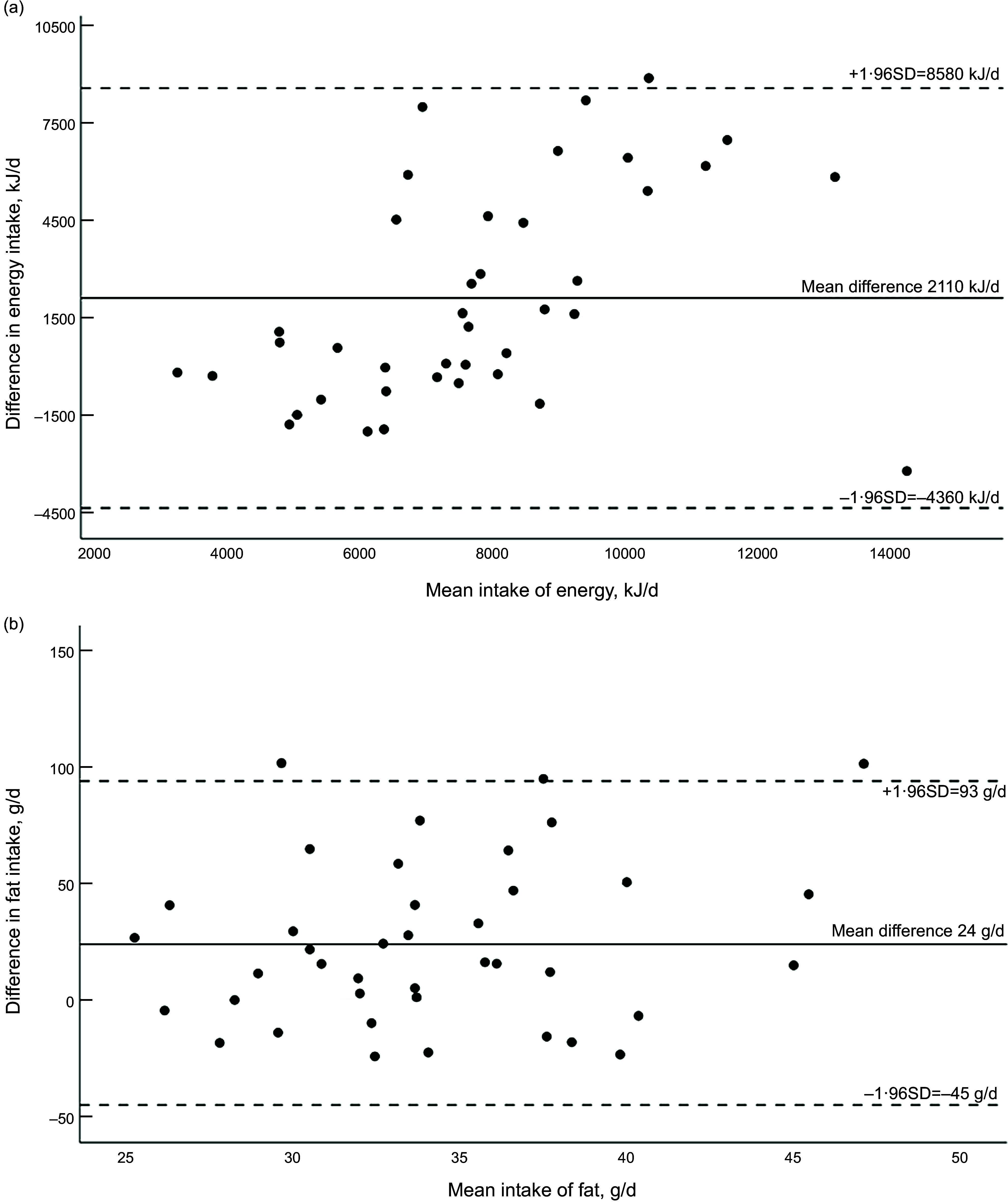
Bland–Altman plot of intake of (a) energy, (b) total fat, from the FFQ and the 24-h recalls. Mean intake on the *x*-axis (mean of FFQ and 24-h recalls) against the difference in intake (FFQ – 24-h recalls) on the *y*-axis, in kJ/d. Dotted lines are limits of agreement (mean difference ± sd × 1·96)

Distributions of the absolute intake estimates of energy and energy-providing nutrients were skewed. The WebFFQasia significantly overestimated the absolute intake of energy, protein, fat and carbohydrates compared with the 24-h dietary recalls (Table 2). The absolute median differences between the methods, at group level, were as follows: 17 %, 15 %, 18 % and 13 % of the 24-h dietary recall estimated intakes, for energy, protein, fat and carbohydrates, respectively. Absolute intakes of sugar, starch and fibre did not differ significantly between the methods (Table 2). Correlations were strong for estimated intakes of saturated fat and moderate for all other energy-providing nutrients, except for total carbohydrate (Table 2). Cross-classification of participants into quartiles of intake showed that correct classifications ranged from 27 % for energy and *n*-3 fatty acids to 48 % for total fat. Misclassification into the opposite quartile ranged from 0 % for saturated fat to 10 % for total carbohydrates (Table 2). BA plots for absolute intakes of energy and total fat intakes are presented in Fig. 1, showing a trend towards increased differences with increasing mean intakes of energy, and wide limits of agreement. For total fat intake differences were evenly distributed above and below the mean intake. Intake of protein and carbohydrates showed the same increasing trends in difference as energy, while fibre, sugar and starch showed the same distributions of differences as total fat intake (Appendix Figures 1A-1E).

**Table 2:** Estimated absolute intakes of energy and energy providing nutrients from the FFQ and the 24-h recalls (*n* 40)

	FFQ		24-h recall			Absolute difference			Cross-classification (%)		
Absolute intake	Median	IQR	Median	IQR	*P* †	Median	IQR	***r*** ‡	Exact	Exact + adj.	Miscl. §
Energy (MJ/d)	8·4	6·0–10·9	6·8	5·6–7·9	0·002	1·1	–0·3–5·2	0·38*	27	73	2
Protein (g/d)	96	63–118	70	58–85	0·002	11	–6–53	0·34*	33	65	7
Fat (g/d)	80	52–114	59	46–70	<0·001	16	–3–47	0·47*	48	80	3
Saturated fat (g/d)	25	17–41	22	11–25	0·001	7	–2–19	0·62*	39	81	0
Monounsatured fat (g/d)	31	20–40	22	17–27	<0·001	6	–1–17	0·31*	37	81	5
Polyunsaturated fat (g/d)	15	10–22	12	9–15	0·006	3	–1–10	0·40*	29	76	2
*n*-3 fatty acids (g/d)	3	2–4	0·002	1–3	0·002	0·6	–0·03–2·0	0·40*	27	71	5
Carbohydrates (g/d)	208	151–305	188	130–207	0·02	23	–27–117	0·29	40	70	10
Sugar	20	9–34	15	7–33	0·4	2	–11–15	0·49*	35	83	7
Starch	126	89–168	109	90–146	0·2	4	–24–53	0·31	38	73	5
Fibre (g/d)	26	20–39	26	20–39	0·05	6	1–6	0·39*	35	78	7

IQR, interquartile range (25th and 75th percentiles); adj., classified in adjacent quartiles; Miscl., misclassification of intake defined as opposite quartiles.

* Statistical significance (*P* < 0·05).

† *P* value by Wilcoxon’s signed-rank test.

‡ Spearman’s rank correlation.

§ Misclassification of intakes defined as opposite quartiles.

When estimating energy percentages (E%), there were no significant differences between the assessment methods for the energy-providing nutrients, except for monounsaturated fat, which the WebFFQasia significantly overestimated compared with the 24-h dietary recalls (Table 3). At a group level, the mean estimates from the FFQ differed with –0·3, 7·0, –4·7, –2·5 and 1·1 E% from the 24-h dietary recall estimates for protein, total fat, carbohydrates, sugar and fibre, respectively. Correlations were strong for E% from sugar and saturated fat and moderate for E% from fibre, carbohydrate, fat and protein. Poor correlations were seen for E% from mono- and polyunsaturated fat. The correct cross-classification ranged from 28 % for E% from polyunsaturated fat to 48 % for E% from total fat. Misclassification in the opposite quartile ranged from 0 % for saturated fat to 10 % for carbohydrates (Table 3). BA plots for the E% estimates showed broad limits of agreement and differences distributed randomly above and below the mean difference for E% from protein and total fat and increasing differences with increasing mean intake of E% from carbohydrates and sugar (Appendix Figures 1F-1I).

**Table 3:** Estimated energy percentage (E%) from energy-providing nutrients, from the FFQ and 24-hour recalls (*n* 40)

	FFQ		24-h recall			Difference			Cross-classification (%)		
Energy percentage	Mean	95 % CI	Mean	95 % CI	*P* †	Mean	95 % CI	*r* ‡	Exact	Exact + adj.	Miscl.
Protein (E%)	18·4	17·2, 19·7	18·5	17·3, 19·7	0·94	–0·06	–1·5, 1·4	0·34*	33	65	8
Fat (E%)	35·3	33·4, 37·2	33·1	30·9, 35,2	0·06	2·22	–0·1, 4·5	0·35*	48	80	3
Saturated fat (E%)	11·7	10·7, 12·7	11·0	9·9, 12·2	0·20	0·65	–0·4, 1·7	0·54*	35	80	0
Monounsaturated (E%)	13·7	12·7, 14·6	12·3	11·4, 13·3	0·04	1·34	0·1, 2·6	0·11	33	78	5
Polyunsaturated (E%)	6·8	6·2, 7·4	6·6	6·0, 7·3	0·63	0·18	–0·6, 0·9	0·20	28	75	3
Carbohydrate (E%)	43·4	41·0, 45·9	45·6	43·1, 48·1	0·13	–2·16	–5·0, 0·7	0·34*	40	70	10
Sugar (E%)§	4·2§	2·6, 7·2||	4·0 §	2·3, 7·7||	0·43¶	–0·80	–2·1, 0·5	0·51*,**	35	83	8
Fibre (E%)	2·7	2·5, 2·9	2·7	2·4, 3·0	0·82	0·03	–0·2, 0·3	0·49*	35	78	8

adj., classified in adjacent quartiles; Miscl., misclassification of intake defined as opposite quartiles.

* Statistical significance (*P* < 0·05 for all analyses).

† *P* value by paired-sample Student’s *t* test.

‡ Pearson’s correlation.

§ Non-parametric distribution, median, 25th and 75th percentiles.

|| Interquartile range.

¶ Wilcoxon’s signed-rank test.

** Spearman’s correlation analysis.

For total energy intake, the results from three analyses showed moderate to good evaluation (Spearman’s correlation, cross-classification and BA plot), while group estimates showed poor evaluation (Wilcoxon’s signed rank test and absolute difference). For absolute protein intake three analyses show moderate (Spearman’s correlation, cross-classification and BA plot) and one showed poor (Wilcoxon’s signed rank test) evaluation, while for protein E% two analyses showed moderate (Spearman’s correlation and BA plot) and two showed good (Wilcoxon’s signed rank test and cross-classification) evaluation. For both absolute intake and E% from total fat, cross-classifications showed good evaluations, and for E% from fat also group intake estimates showed good evaluation. For total carbohydrate intake, three analyses showed poor evaluation and one (BA plot) showed moderate evaluation. For E% from carbohydrates, group estimate showed good evaluation (Wilcoxon’s signed rank test), two analyses showed moderate evaluations (Spearman’s correlation and BA plot), while the cross-classification showed poor evaluation.

Individual differences between methods for energy, protein, fat and carbohydrates are further visualised in Appendix Figures 3A–3D.

#### Intake of vitamins and minerals

The WebFFQasia significantly overestimated the absolute intake of all micronutrients except vitamin D, Fe and Na, compared with the 24-h dietary recalls (Table 4). Correlations were strong for thiamine, niacin, folic acid, vitamin B_12_, vitamin E, Zn and Se. There were moderate correlations for Mg, Na, iodine, phosphorus, Ca and vitamins A, B_3_, B_6_, C and D, and poor correlations were observed for Fe and potassium. Cross-classification into the correct quartile ranged from 30 % for Na to 50 % for vitamin B_6_. Misclassification in the opposite quartile ranged from 0 % for thiamine, Na, Se and iodine to 8 % for Fe, Ca and phosphorous (Table 4). A total of nine participants registered no use of dietary supplements in the WebFFQasia. Of these, four registered intake of dietary supplements and five did not register intake of dietary supplements in the 24 h recalls.

**Table 4:** Micronutrient intake estimated from the FFQ and the 24-hour recalls (*n* 40)

	FFQ		24-hour recall		Absolute difference				Cross-classification		
Absolute intake	Median	IQR	Median	IQR	Mean	sd	*P* †	*r* ‡	Exact	Exact + adj.	Miscl.
Vitamin A (*µ*g/d)	744	530–1349	480	322–631	447	469	<0·001	0·37*	38	73	5
Vitamin B_1_ (mg/d)	1·9	1·3–3·5	1·4	1·0–2·4	0·6	1·1	<0·01	0·54**	38	83	0
Vitamin B_2_ (mg/d)	2·1	1·5–3·6	1·6	1·1–2·9	0·6	1·2	<0·01	0·52**	38	83	3
Vitamin B_3_ (mg/d)	25·9	16·2–38·7	20·3	13·5–30·0	6·7	13·8	<0·01	0·48**	43	83	3
Vitamin B_6_ (mg/d)	2·5	1·5–3·3	1·9	1·4–2·6	0·5	1·2	<0·01	0·45**	50	75	3
Folic acid (*µ*g/d)	388	238–649	275	194–498	47·0	3–241	<0·01	0·52**	48	83	3
Vitamin B_12_ (*µ*g/d)	6·5	4·3–9·8	5·0	3·2–6·8	2·0	3·1	<0·01	0·54**	33	90	3
Vitamin C (mg/d)	149	105–213	75	47–117	76	127	<0·001	0·34*	33	75	3
Vitamin D (mg/d)	13	7–22	16	5–30	−5·9	30·5	0·77	0·40	33	78	5
Vitamin E (mg/d)	22	13–29	14	11–20	8·3	11·7	<0·001	0·53	40	88	3
Iron (mg/d)	12·0	8·4–20·3	12·0	7·6–21·9	−2·1	27·7	0·91	0·27	40	75	8
Calcium (mg/d)	809	441–1249	674	448–919	251	607	<0·01	0·42**	45	85	8
Magnesium (mg/d)	370	267–469	292	225–404	72	140	<0·01	0·45**	43	85	3
Sodium (g/d)	2·2	1·5–2·7	2·0	1·5–2·8	0·1	1·1	0·56	0·36	30	73	0
Potassium (g/d)	3·9	2·7–5·3	2·7	2·0–3·1	1·4	1·4	<0·001	0·27	33	78	5
Phosphorus (g/d)	1·7§	1·5, 2·0	1·3§	1·2, 1·4	0·4	0·7	<0·01||	0·37¶	40	73	8
Zinc (mg/d)	13·0	9·3–17·0	9·7	7·4–15·9	2·2	5·9	0·03	0·64**	40	88	3
Selenium A (*µ*g/d)	71·5	48·0–88·8	59·0	41·5–82·5	12·8	31·1	0·02	0·61**	45	88	0
Iodine A (*µ*g/d)	220	160–337	110	76–169	104	102	<0·001	0·45**	33	75	0

IQR, interquartile range (25th and 75th percentiles); adj., Classified in adjacent quartiles; Miscl., misclassification of intake defined as opposite quartiles.

* Correlation is significant at the 0·05 level.

** Correlation is significant at the 0·01 level.

† *P* value by Wilcoxon’s signed-rank test.

‡ Spearman’s correlation analysis.

§ Parametric distribution, mean and 95 % CI.

|| Paired-sample Student’s *t* test.

¶ Pearson’s correlation analysis.

#### Intake of food and beverages

Intake of food and beverages in grams per day is presented in Table 5. The food intakes from the WebFFQasia were not significantly different from the 24-h dietary recall estimates for fourteen out of twenty-three food groups. Estimated intakes of cakes, cereals, rice and pasta, legumes, meat, egg, yoghurt, cheese, butter and margarine, sugar and sweets, all beverages and snacks showed no significant differences between methods. Intakes of bread, potato and potato products, vegetables (not including legumes), fruit and berries, nuts and seeds, fish and milk and cream were significantly overestimated by the WebFFQasia. Median intake of spices and herbs was significantly underestimated by the FFQ by 2 g/d.

**Table 5:** Estimated intakes of food and beverages from the FFQ and 24-h recalls (g/d)

	FFQ (g/d)		24 h recall (g/d)			Difference (g/d)‡			Cross-classification		
Food group	Median	IQR	Median	IQR	*P* †	Mean	Median	*r* §	Exact	Exact + adj.	Miscl.
Bread, crispbread	115	67–180	87	58–136	0·04	51	31	0·26	45	68	8
Cakes and sweet pastries	25	9–51	19	7–45	0·30	5	1	0·55**,||	43	70	3
Cereals, rice, pasta	51	25–92	68	37–117	0·09	–29	–12	0·33*	38	73	5
Potatoes and potato prod.	23	11–73	9	2–30	<0·01	24	10	0·15	23	70	10
Legumes	14	7–30	9	0–40	0·14	6	4	0·79**,||	33	58	10
Other vegetables	256	159–382	150	115–198	<0·01	137	133	0·08	40	83	8
Fruit and berries	144	87–332	54	30–109	<0·01	134	99	0·31	33	73	5
Fruit juices	22	6–75	0	0–51	<0·01	44	8	0·14	28	78	5
Nuts and seeds	10	4–30	3	0–10	<0·01	14	5	0·43**	40	73	8
Meat	83	36–177	62	11–151	0·12	25	20	0·41**	33	70	3
Fish	61	12–115	18	0–45	<0·01	37	26	0·44**	43	80	10
Egg	33	14–50	18	0–41	0·05	14	4	0·41**	38	73	3
Milk, cream	12	8–21	0	0–10	<0·01	8	10	0·14	35	75	8
Yoghurt	180	45–392	169	81–358	0·38	124	–6	0·36*	48	78	10
Cheese	12	8–25	17	9–32	0·16	–5	–3	0·51**	45	83	3
Butter and margarine	24	15–44	22	8–29	0·07	9	7	0·23	20	68	3
Sugar and sweets	11	5–23	8	2–16	0·28	–4	1	0·41**	25	70	3
Beverages, total	1500	981–2051	1192	993– 1894	0·29	113	139	0·45**	30	78	3
Drinking water	919	706–1436	800	558–1354	0·42	47	10	0·41**	40	78	5
Coffee	37	3–250	25	0–136	0·06	72	3	0·75**,||	43	80	0
Tea	74	8–375	154	62–385	0·12	–66	–22	0·33*	33	73	3
Snacks	2	1–7	0	0–4	0·38	–1	1	0·09	23	68	0
Spices and herbs	1·6	1–2	2·3	1–5	<0·01	–2	–1	0·40*	38	75	3

IQR, interquartile range (25th and 75th percentiles); adj.: classified in adjacent quartiles Miscl., misclassification of intake defined as opposite quartiles.

* *P* < 0·05.

** *P* < 0·05.

† Non-parametric test.

‡ FFQ-24-h recall.

§ Spearman’s correlation.

|| Pearson’s correlation.

For cakes, legumes, cheese and coffee, correlations between methods were strong. Of the remaining nineteen food groups, moderate correlations were observed for twelve and poor correlations for seven categories. Misclassification in the opposite quartile ranged from 0 % for coffee and snacks to 10 % for potato and potato products, legumes, fish and fish products and yoghurt (Table 5).

Intakes of all food groups except bread, other cereals, cakes and vegetables showed increasing differences between methods with increasing mean intake (Appendix Figures 2A–2H). Of these, fish, fruits and berries, juices, nuts and seeds showed tendencies towards increased overestimation with increased mean intake, tea and spices showed the opposite and the remaining food groups showed increasing differences randomly distributed above and below the average difference.

Out of the forty participants, nine answered ‘no’ to the initial question in the WebFFQasia of whether their diet included ‘mostly South Asian dishes’, and hence did not answer the questions about these specific dishes. However, according to the 24-h dietary recalls, six of these participants did register intake of South Asian dishes. There were no significant differences in energy intake between the participants who answered ‘Yes’ and ‘No’ to the ‘mostly South Asian dishes’ question (results not shown).

## Discussion

The present study evaluated estimated intakes of energy, nutrients and food groups, assessed using an extended digital FFQ for women of South Asian origin living in Norway. Evaluated against the 24-h dietary recall reference method, the absolute intakes of energy and energy-providing nutrients were overestimated by the WebFFQasia. However, when calculated as E%, no significant differences were observed, except for monounsaturated fat. The WebFFQasia thus shows a good ability to assess E% at the group level. The ability to rank the participants according to E% was also good to adequate for E% from protein, total fat, total carbohydrates, sugar and fibre and poor for MUFA and PUFA.

### Energy and energy-providing nutrients

The overall evaluation suggests clear trends of overestimation of energy from the WebFFQasia compared with the 24-h recalls. An earlier validation study showed that the energy intake estimated using the original web-FFQ did not differ significantly, at the group level, from total energy expenditure assessed using the doubly labelled water method^(13)^. In the same validation study, the estimated energy intake from 24-h dietary recalls was significantly underreported compared with the total energy expenditure assessments from the doubly labelled water. In the present study, the energy intake from the WebFFQasia was higher than the energy intake from the 24-h dietary recalls, as seen in the validation study from 2017^(13)^. If the difference between energy intake from the WebFFQasia and the 24-h dietary recall was partly affected by possible underreporting of the 24-h dietary recalls, this is unknown but the results from the doubly labelled water study of 2017 may suggest this. However, as shown in both studies, the large individual variations in E% estimates from the two methods underline the importance of using the WebFFQasia to estimate intakes at the group level and that individual estimates from the WebFFQasia must be used with caution and a strong awareness of the uncertainty of the estimates at an individual level.

Except for MUFA and PUFA, the evaluation of E% from the energy-providing nutrients showed, overall, good results, for both mean intakes at the group level and correlation and classification across quartiles of intake. This agrees with the results from both of the two earlier studies^(7,13)^, which showed overestimation of absolute intakes of macronutrients, whereas the E% from the energy-providing nutrients agreed with the reference methods. This therefore suggests that the present WebFFQasia can adequately assess E% at the group level in this population. Based on the correlation and cross-classification results, the WebFFQasia can rank the participants adequately for most macronutrients, except for MUFA, similar to the results from the evaluation study of the non-modified web-FFQ in South Asians^(7)^.

### Vitamins and minerals

Most absolute intakes of vitamins and minerals were overestimated by the WebFFQasia. The exceptions were intakes of vitamin D, Fe and Na. This agrees partly with the earlier evaluation, which also found good agreement between the web-FFQ and the reference method for Fe and Na^(7)^. In the earlier study, there was also good agreement for the estimates of vitamin E and Se, which, in the present evaluation, showed significant differences between methods. These differences in results may have random causes or be explained by the added questions about South Asian dishes where vegetable oils, sources of vitamin E in the diet, are important ingredients and also used widely for cooking. Variations between the amounts of vegetable oils used in the standard recipes of the food composition database and individual variations in use among the participants while cooking may have influenced the results.

In the present evaluation study, good ranking ability was observed for vitamin B_1_, vitamin B_2_, folic acid, vitamin B_12_, vitamin E, Zn and Se, and adequate ranking was seen for the remaining investigated micronutrients, except potassium and Fe. Thus, the WebFFQasia may be used to rank participants according to intakes of vitamin and minerals; however, results for Fe and potassium should be used with caution.

### Food groups

In the present evaluation, the WebFFQasia showed a good ability to estimate absolute intake of food groups at the group level, for fourteen of the twenty-three food groups, whereas intakes of bread, milk and cream, fruit, berries, nuts, potatoes, vegetables, not including legumes, and fish were all overestimated at the group level compared with the reference method. This agrees partly with the findings of Medin et al. also showing overestimation of potatoes, fruit and vegetables, fish, milk and cream in the original web-FFQ^(13)^.

The correlations and cross-classification results showed moderate-to-good agreement, and the WebFFQasia may thus be used to rank participants according to the main food groups, but should, however, be used with some caution for estimates of potato, potato products, vegetables (not legumes) and snack intakes.

### Study population

In the present study with women of South Asian origin in Norway, we met challenges related to recruitment and data collection. Quay and colleagues^(6)^ identified factors that could facilitate recruitment and data collection in dietary surveys among South Asians in the UK, including culture-specific assessment tools, visual aid reinforcement and the involvement of key community members and translators. In the present study, we applied an online, web-based FFQ that was designed to give better coverage of South Asian diets and common South Asian dishes. We used visual aids with regard to portion sizes and a dietitian who spoke Urdu and Hindi and was familiar with South Asian diet culture conducted the 24-h dietary recalls. A total of 68 % of the study population had education at university level. This is a higher share than observed for the general population of women with South Asian origin in Norway, which is approximately 38 % (statistics Norway, accessed November 2023, ssb.no/en/statbank/), and this may influence the results.

### Strengths and limitations

The strengths of the study include the use of culture-specific assessment tools, 24-h dietary recalls being conducted in Urdu or Hindi if necessary and the interviewer having knowledge of Asian diet and culture. These measures may have reduced the participation burden, as pointed out by Quay and colleagues^(6)^.

All self-reporting dietary assessment methods are prone to measurement errors. Misreporting of food and energy intake using self-reported dietary assessment methods has been extensively reported^(17–21)^. The inherent limitations of the closed food lists, the abstraction of frequencies and the standard portion sizes of an FFQ make it a difficult cognitive task for respondents and challenge their memory^(22)^. Underreporting of food intake when diet is assessed using 24-h recalls has been seen to be associated with both observational and reporting effects^(20)^. Also, due to the design of 24-h recalls, the method may be prone to underestimate intake of foods that are eaten infrequently or seldom. In an evaluation study, such as the present one, evaluation of an FFQ designed for assessing average habitual diet over the last year, with frequency alternatives including infrequent intakes, using 24-h recalls as the reference method, may result in a more uncertain evaluation of infrequently and rarely eaten food items and the associated micronutrient intakes. Some of the poor agreement between the methods could be due partly to 24-h dietary recalls not capturing less frequent intakes, compared with the FFQ which covers average intake over the last year. This may explain the poor agreement observed between estimated intakes of fish, a food group many eat infrequently and seldom. Hence, disagreement between the methods may result from errors in both methods and suggests that the assessment of certain seldom eaten food items should be evaluated against other dietary assessment methods that capture rare intakes better than 24-h dietary recalls^(22)^.

The small sample size is a limitation of this study, and a study sample of at least fifty, preferably larger is desirable^(11)^. This will have affected the analyses and the outcomes of the study, making the results less robust. However, the evaluation study was performed in a sub-group of the main study population, which is a strength as age, ethnic group, gender and health status of the population also may affect the results of an evaluation study^(11)^.

### Conclusions

The present evaluation of the WebFFQasia, an FFQ designed to assess the habitual diet of South Asian women living in Norway, showed that it may be used to assess E% from habitual diet and can adequately estimate intakes and rank participants according to the intakes of the nutrients and main food categories at the group level. The dietary assessment tool is designed for group-level estimates and should not be used for individual dietary assessments due to the high level of individual measurement errors. Future studies applying the WebFFQasia for dietary assessment may reflect on a potential tendency of overreporting absolute intakes of fruit, vegetables and fish.

## Supporting information

Carlsen et al. supplementary material Carlsen et al. supplementary material

## Supplementary material

To view supplementary material for this article, please visit https://doi.org/10.1017/S1368980024000302
