# Supplementary material for: Evaluation of a digital FFQ using 24 h recalls as reference method, for assessment of habitual diet in women with South Asian origin in Norway
Source: Public Health Nutr. 2024 Feb 6;27(1):e55. doi: 10.1017/S1368980024000302 (PMC10882527; doi:10.1017/S1368980024000302)
Supplement: Carlsen et al. supplementary material [file S1368980024000302sup001.docx]

Appendix to the manuscript Carlsen et al. Evaluation of a digital FFQ using 24 hour recalls as reference method, for assessment of habitual diet in women with South Asian origin in Norway

Figures


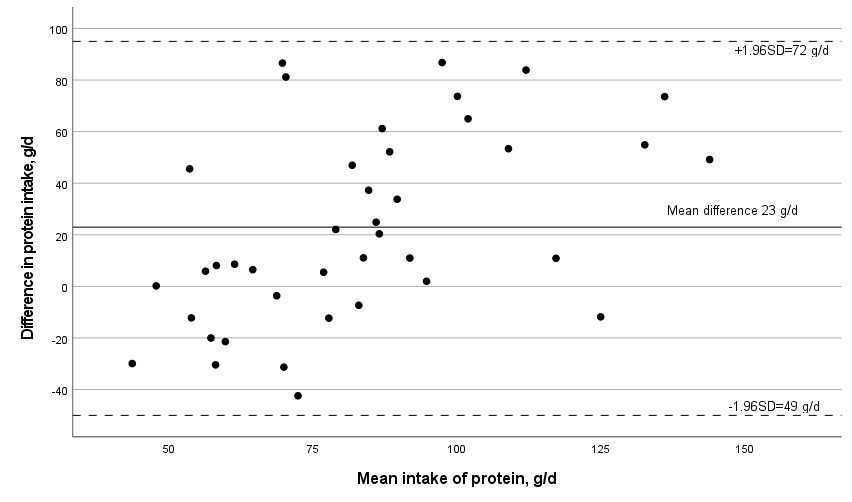


Appendix Figure 1A. Bland–Altman plot of the intake of protein from the food frequency questionnaire (FFQ) and the 24-hour recalls. Mean intake on the *x* axis (mean of FFQ and 24-hour recalls) against the difference in intake (FFQ – 24-hour recalls) on the *y* axis, in g/day. Dotted lines are limits of agreement (mean error ± standard deviation × 1.96).


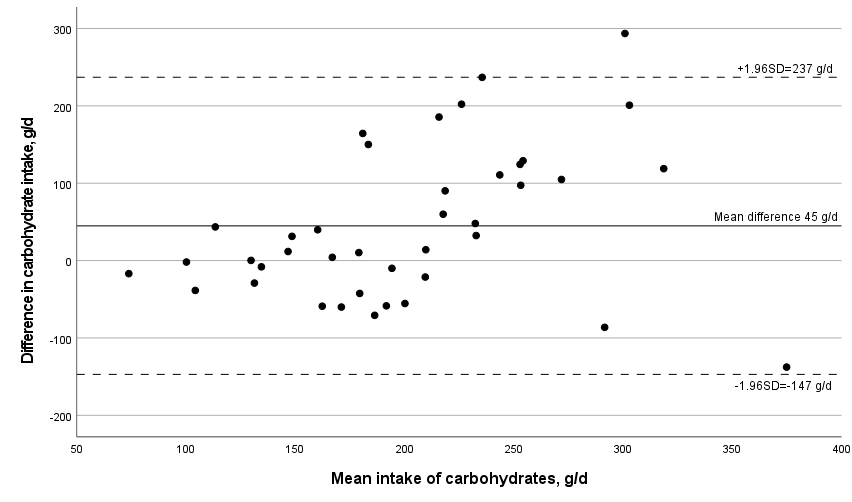


Appendix Figure 1B. Bland–Altman plot of the intake of carbohydrates from the food frequency questionnaire (FFQ) and the 24-hour recalls. Mean intake on the *x* axis (mean of FFQ and 24-hour recalls) against the difference in intake (FFQ – 24-hour recalls) on the *y* axis, in g/day. Dotted lines are limits of agreement (mean error ± standard deviation × 1.96).


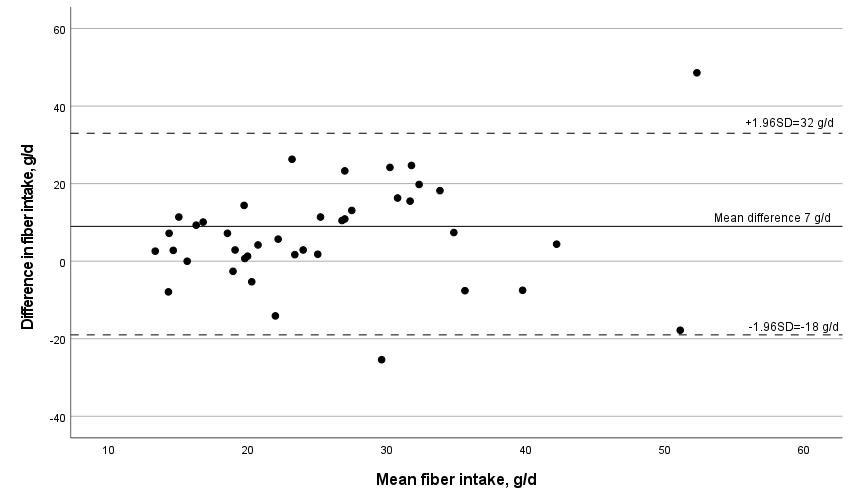


Appendix Figure 1C. Bland–Altman plot of the intake of fiber from the food frequency questionnaire (FFQ) and the 24 hour recalls. Mean intake on the *x* axis (mean of FFQ and 24-hour recalls) against the difference in intake (FFQ – 24-hour recalls) on the *y* axis, in g/day. Dotted lines are limits of agreement (mean error ± standard deviation × 1.96).


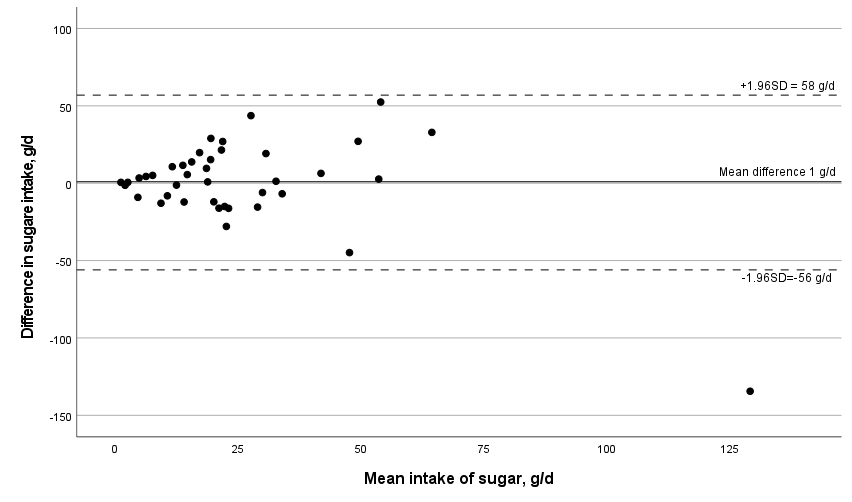


Appendix Figure 1D. Bland–Altman plot of the intake of sugar from the food frequency questionnaire (FFQ) and the 24-hour recalls. Mean intake on the *x* axis (mean of FFQ and 24-hour recalls) against the difference in intake (FFQ – 24-hour recalls) on the *y* axis, in g/day. Dotted lines are limits of agreement (mean error ± standard deviation × 1.96).


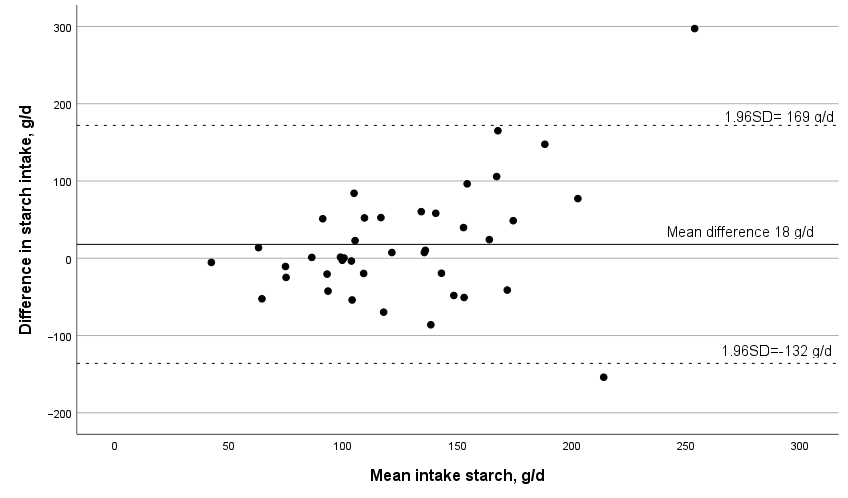


Appendix Figure 1E Bland–Altman plot of the intake of starch from the food frequency questionnaire (FFQ) and the 24-hour recalls. Mean intake on the *x* axis (mean of FFQ and 24-hour recalls) against the difference in intake (FFQ – 24-hour recalls) on the *y* axis, in g/day. Dotted lines are limits of agreement (mean error ± standard deviation × 1.96).


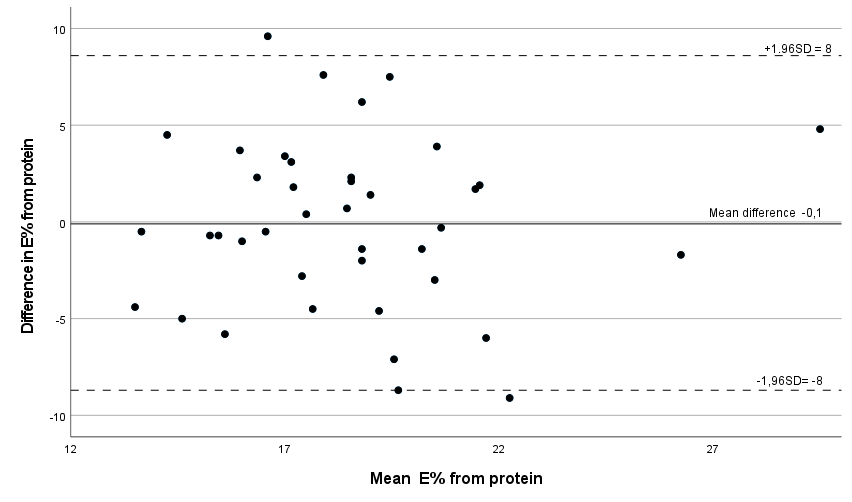


Appendix Figure 1F Bland–Altman plot of the intake of E% from protein, from the food frequency questionnaire (FFQ) and the 24-hour recalls. Mean intake on the *x* axis (mean of FFQ questionnaire and 24-hour recalls) against the difference in intake (FFQ – 24-hour recalls) on the *y* axis, in E% per day. Dotted lines are limits of agreement (mean error ± standard deviation × 1.96).


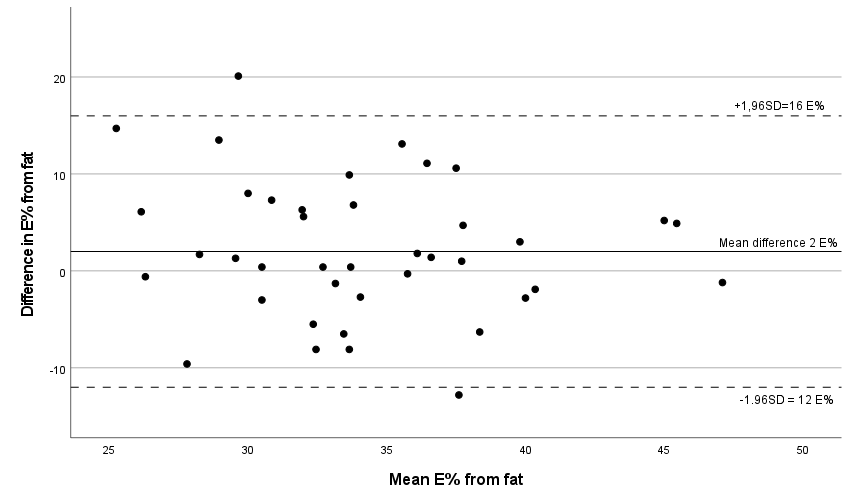


Appendix Figure 1G Bland–Altman plot of the intake of E% from fat, from the food frequency questionnaire (FFQ) and the 24-hour recalls. Mean intake on the *x* axis (mean of FFQ and 24-hour recalls) against the difference in intake (FFQ – 24-hour recalls) on the *y* axis, in E% per day. Dotted lines are limits of agreement (mean error ± standard deviation × 1.96).


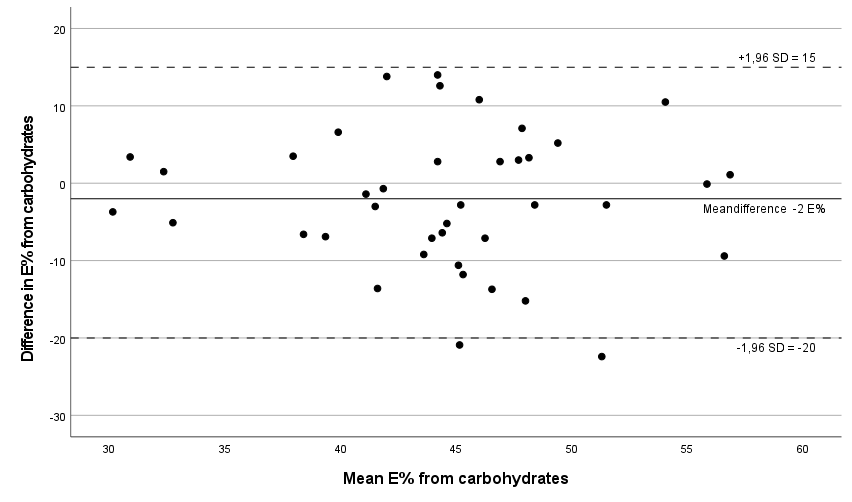


Appendix Figure 1H Bland–Altman plot of the intake of E% from carbohydrates, from the food frequency questionnaire (FFQ) and the 24-hour recalls. Mean intake on the *x* axis (mean of FFQ and 24-hour recalls) against the difference in intake (FFQ – 24-hour recalls) on the *y* axis, in E% per day. Dotted lines are limits of agreement (mean error ± standard deviation × 1.96).


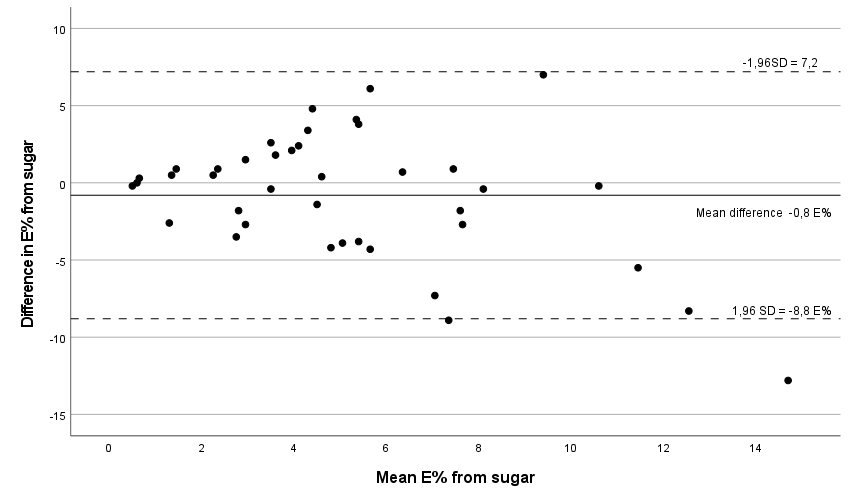


Appendix Figure 1I Bland–Altman plot of the intake of E% from sugar, from the food frequency questionnaire (FFQ) and the 24-hour recalls. Mean intake on the *x* axis (mean of FFQ and 24-hour recalls) against the difference in intake (FFQ – 24-hour recalls) on the *y* axis, in E% per day. Dotted lines are limits of agreement (mean error ± standard deviation × 1.96).


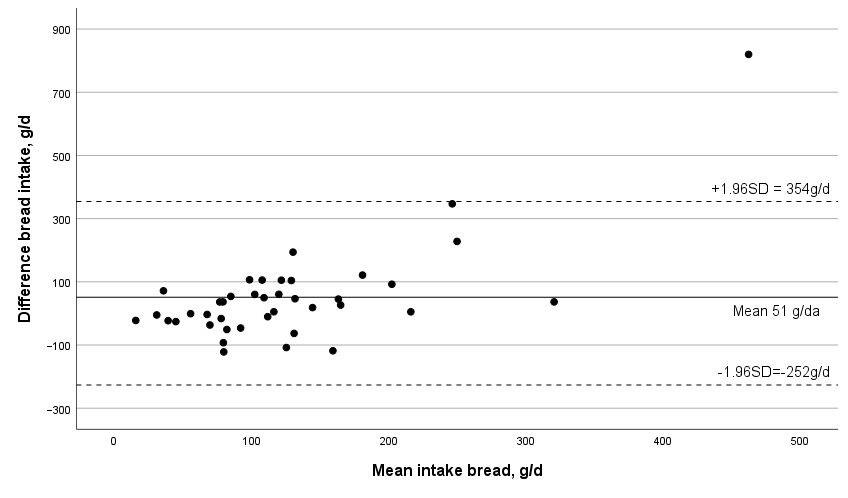


Appendix Figure 2A Bland–Altman plot of the intake of bread and crisp bread from the food frequency questionnaire (FFQ) and the 24-hour recalls. Mean intake on the *x* axis (mean of FFQ and 24-hour recalls) against the difference in intake (FFQ – 24-hour recalls) on the *y* axis, in g/day. Dotted lines are limits of agreement (mean error ± standard deviation × 1.96).


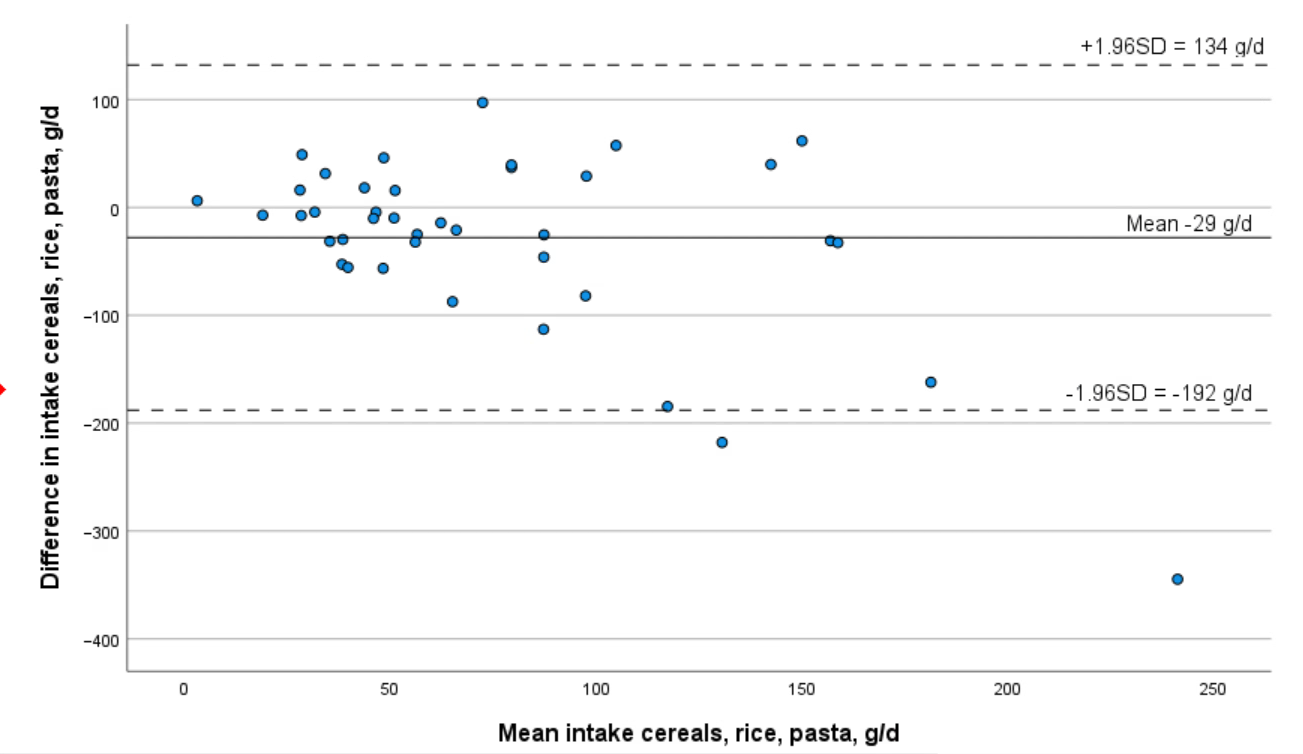


Appendix Figure 2B Bland–Altman plot of the intake of cereals, including rice and pasta, from the food frequency Questionnaire (FFQ) and the 24-hour recalls. Mean intake on the *x* axis (mean of FFQ and 24-hour recalls) against the difference in intake (FFQ – 24-hour recalls) on the *y* axis, in g/day. Dotted lines are limits of agreement (mean error ± standard deviation × 1.96).


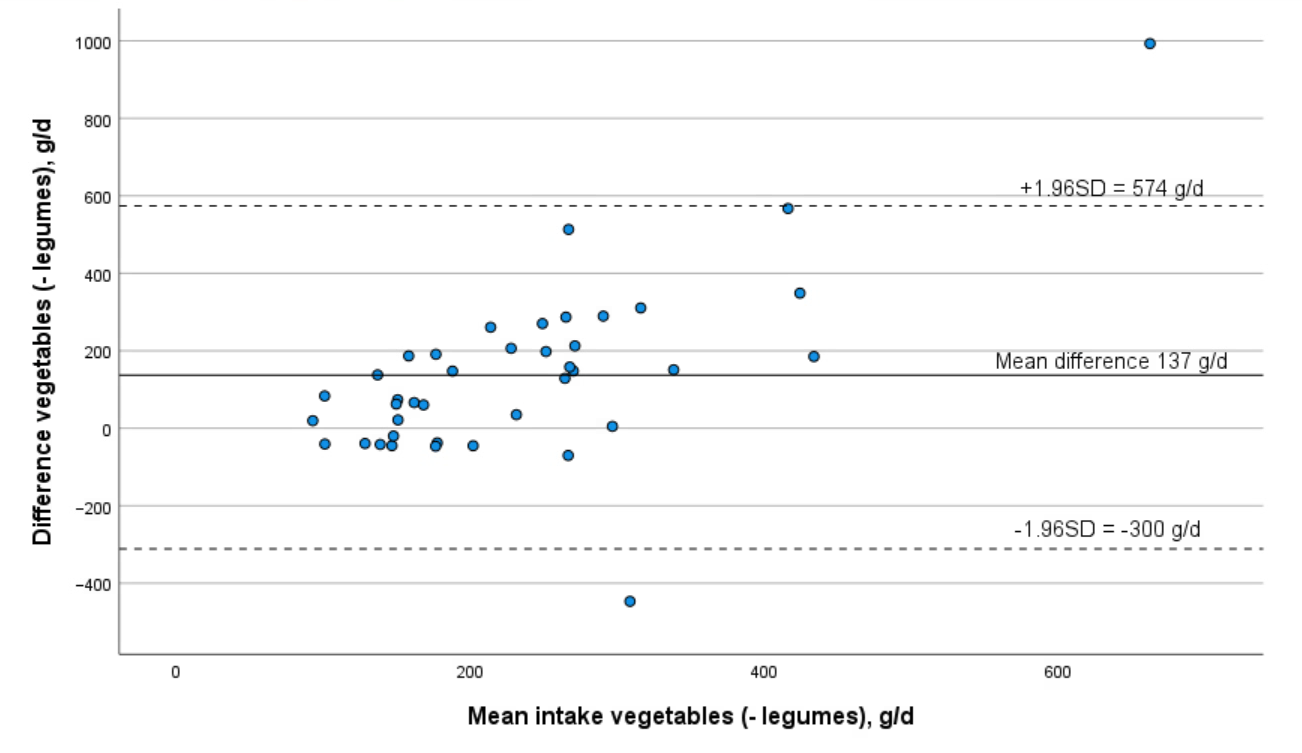


Appendix Figure 2C Bland–Altman plot of the intake of vegetables excluding legumes, from the food frequency questionnaire (FFQ) and the 24-hour recalls. Mean intake on the *x* axis (mean of FFQ and 24-hour recalls) against the difference in intake (FFQ – 24-hour recalls) on the *y* axis, in g/day. Dotted lines are limits of agreement (mean error ± standard deviation × 1.96).


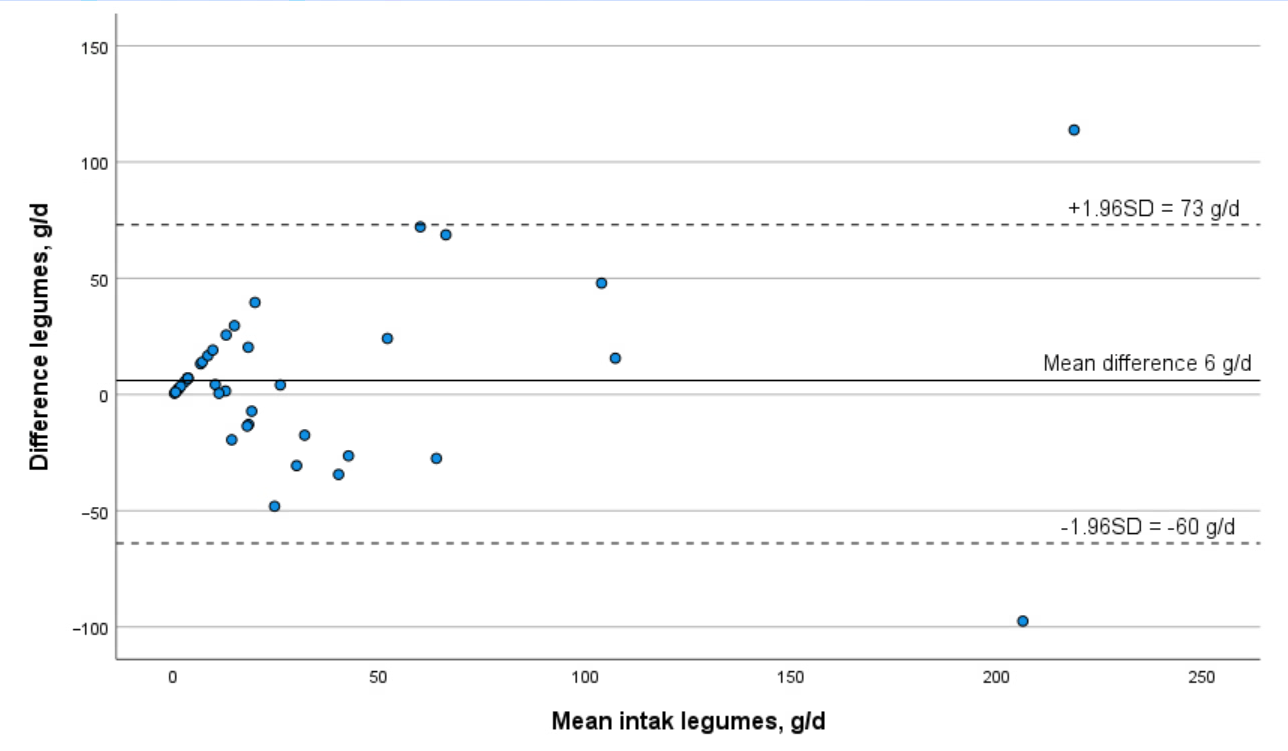


Appendix Figure 2D Bland–Altman plot of the intake of legumes, from the food frequency questionnaire (FFQ) and the 24-hour recalls. Mean intake on the *x* axis (mean of FFQ and 24-hour recalls) against the difference in intake (FFQ – 24-hour recalls) on the *y* axiss, in g/day. Dotted lines are limits of agreement (mean error ± standard deviation × 1.96).


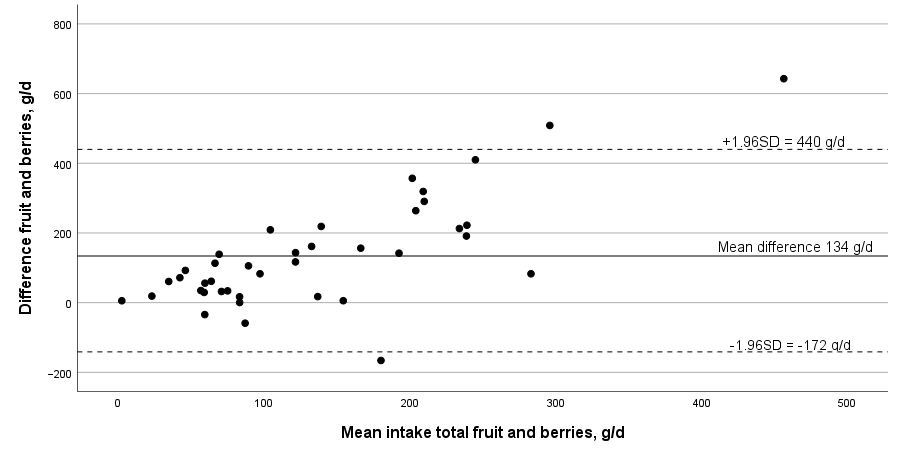


Appendix Figure 2E Bland–Altman plot of the total intake of fruit and berries from the food frequency questionnaire (FFQ) and the 24-hour recalls. Mean intake on the *x* axis (mean of FFQ and 24-hour recalls) against the difference in intake (FFQ – 24-hour recalls) on the *y* axis, in g/day. Dotted lines are limits of agreement (mean error ± standard deviation × 1.96).


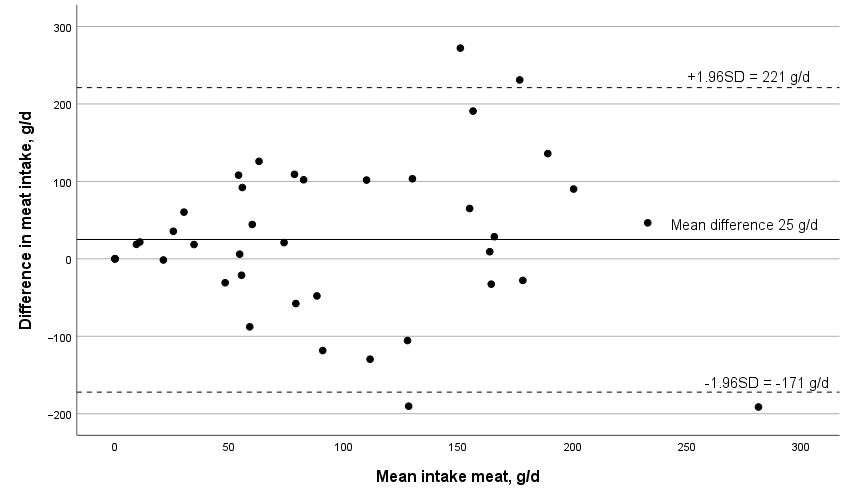


Appendix Figure 2F Bland–Altman plot of the total intake of meat from the food frequency questionnaire (FFQ) and the 24-hour recalls. Mean intake on the *x* axis (mean of FFQ and 24-hour recalls) against the difference in intake (FFQ – 24-hour recalls) on the *y* axis, in g/day. Dotted lines are limits of agreement (mean error ± standard deviation × 1.96).


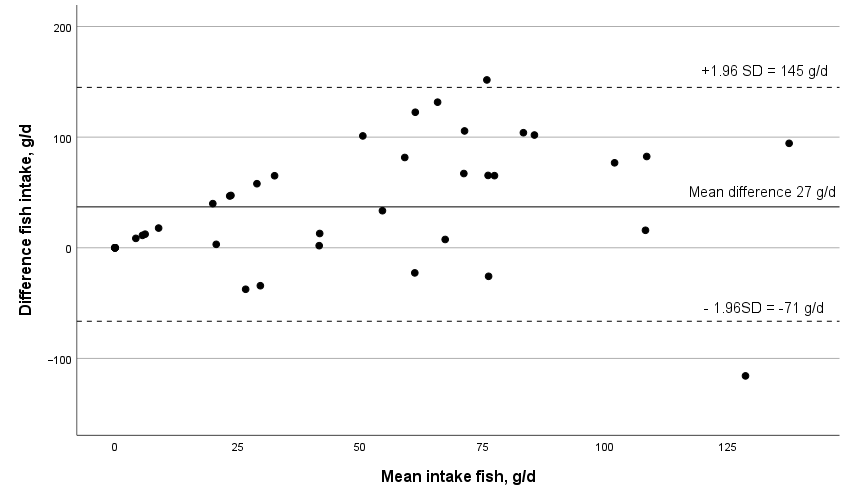


Appendix Figure 2G Bland–Altman plot of the total intake of fish from the food frequency questionnaire (FFQ) and the 24-hour recalls. Mean intake on the *x* axis (mean of FFQ and 24-hour recalls) against the difference in intake (FFQ – 24-hour recalls) on the *y* axis, in g/day. Dotted lines are limits of agreement (mean error ± standard deviation × 1.96).


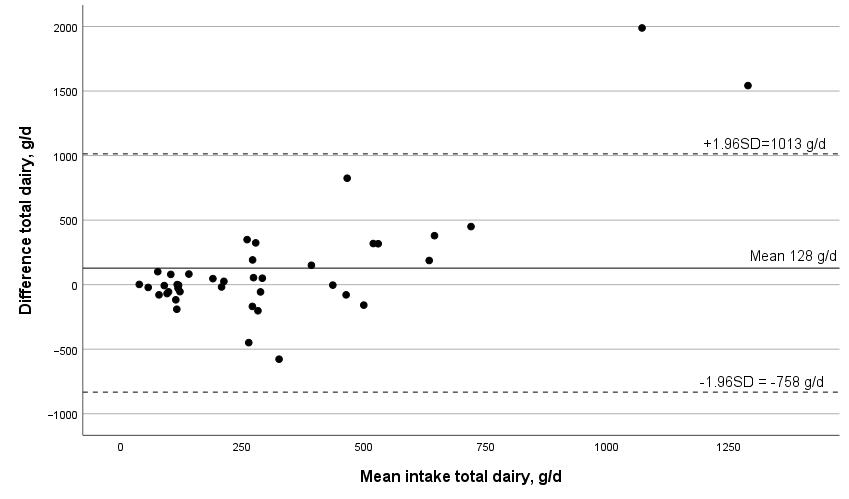


Appendix Figure 2H Bland–Altman plot of the total intake of dairy products from the food frequency questionnaire (FFQ) and the 24-hour recalls. Mean intake on the *x* axis (mean of FFQ and 24-hour recalls) against the difference in intake (FFQ – 24-hour recalls) on the *y* axis, in g/day. Dotted lines are limits of agreement (mean error ± standard deviation × 1.96).


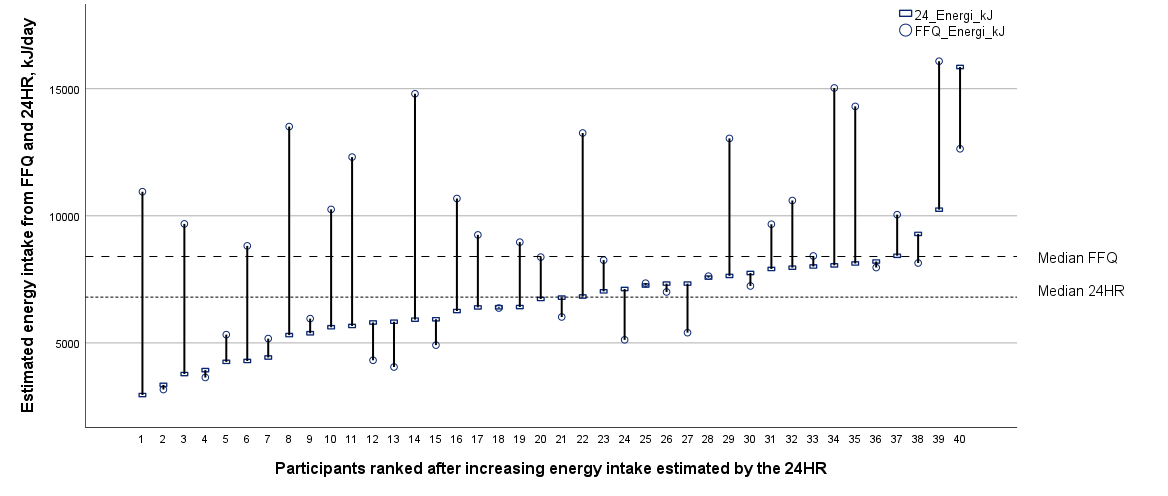


Appendix Figure 3A Drop-line graph of intake of energy, from the 24-hour recalls (24HR; rectangles) and the food frequency questionaire (FFQ; circles). Participants ranked after estimated energy intake from the 24-hour recalls.


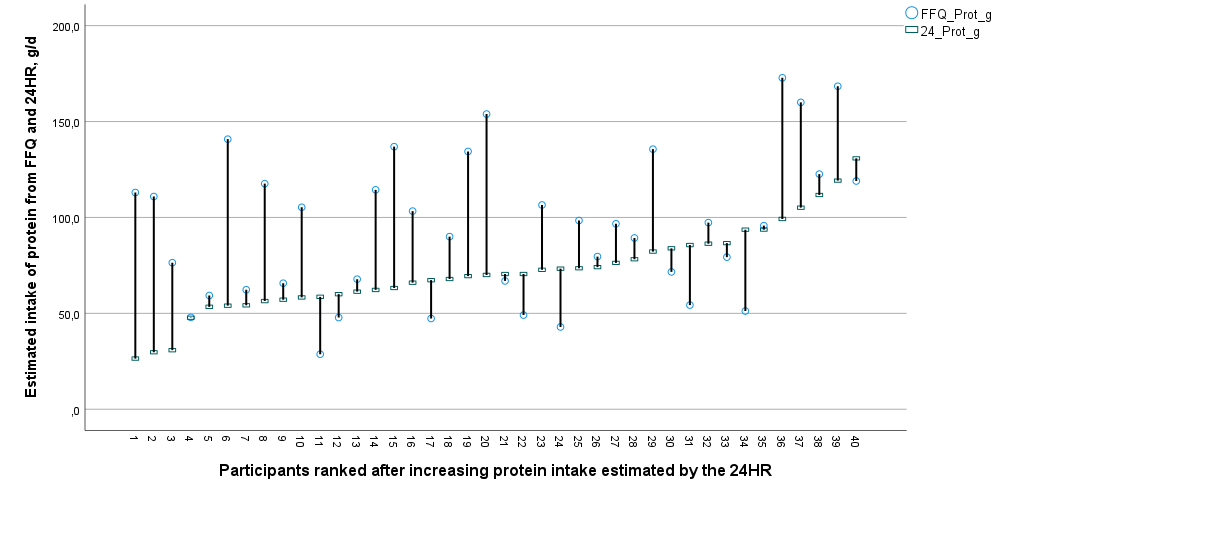


Appendix Figure 3B Drop-line graph of absolute intake of protein, from the 24-hour recalls and the food frequency questionnaire, ranked after increasing intake estimates from the 24-hour recalls.


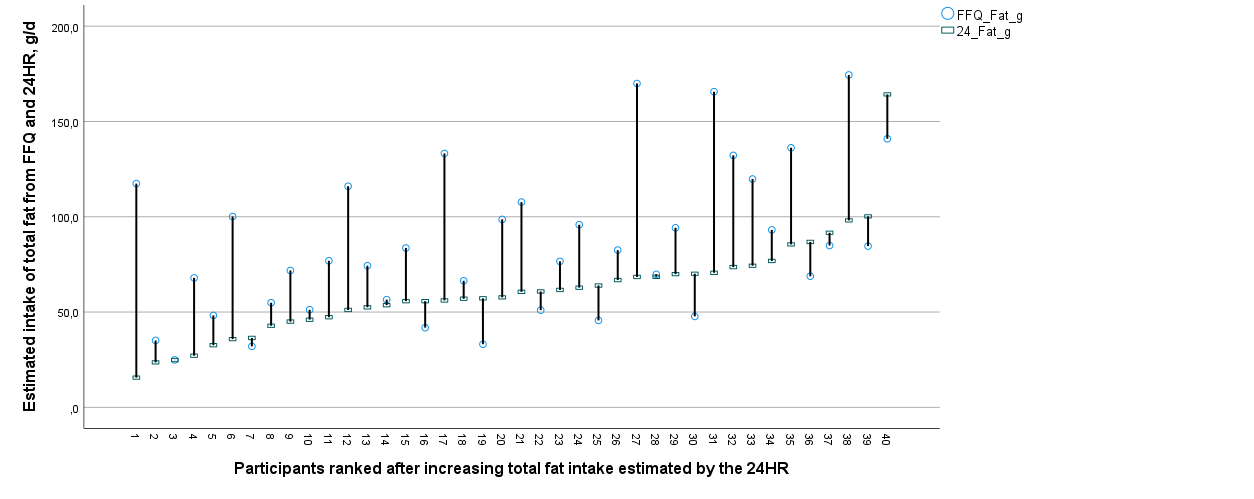


Appendix Figure 3C Drop-line graph of absolute intake of total fat, from the 24-hour recalls and the food frequency questionnaire, ranked after increasing intake estimates from the 24-hour recalls.


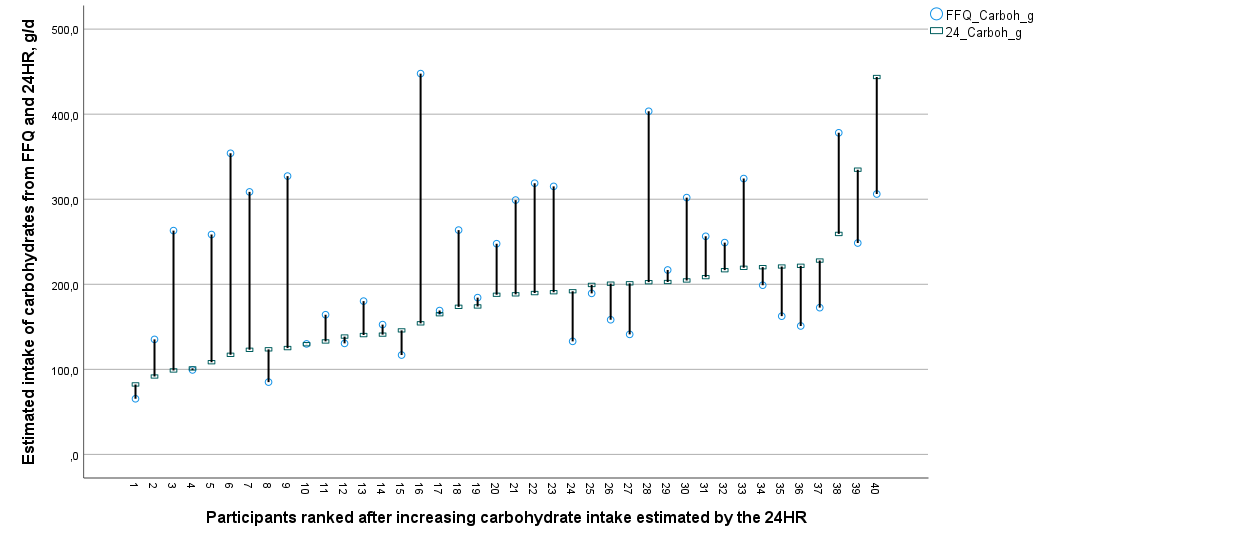


Appendix Figure 3D Drop-line graph of absolute intake of total carbohydrates, from the 24-hour recalls and the food frequency questionnaire, ranked after increasing intake estimates from the 24-hour recalls.
